# Secondary prevention by striking the balance in 24-hour movement behaviour by empowering people at risk with a stroke: rationale and design of the RISE intervention randomised controlled trial

**DOI:** 10.1136/bmjopen-2024-094894

**Published:** 2025-06-05

**Authors:** Camille F M Biemans, Yvonne A W Hartman, Suzanne Broers, Sophie Pagen, Wendy Hendrickx, RISE Study Group, Johanna M van Dongen, Olaf W Verschuren, Coralie English, Cindy Veenhof, Johanna M A Visser-Meily, Martijn F Pisters

**Affiliations:** 1 Department of Rehabilitation, Physiotherapy Science and Sport, University Medical Centre Utrecht Brain Centre, Utrecht, The Netherlands; 2 Department of Health Innovations and Technology, School of Health Sciences, Fontys University of Applied Sciences Physiotherapy, Eindhoven, The Netherlands; 3 Centre for Physiotherapy Research and Innovation in Primary Care, Leidsche Rijn Julius Health Care Centres, Utrecht, The Netherlands; 4 Department of Health Sciences, Faculty of Science, Amsterdam Movement Sciences, Vrije Universiteit Amsterdam, Amsterdam, The Netherlands; 5 Department of Health Sciences, Faculty of Science, Amsterdam Public Health, Vrije Universiteit Amsterdam, Amsterdam, The Netherlands; 6 Centre of Excellence for Rehabilitation Medicine, Brain Centre, University Medical Centre Utrecht and De Hoogstraat Rehabilitation, Utrecht, The Netherlands; 7 School of Health Sciences, University of Newcastle, Newcastle, New South Wales, Australia; 8 Research Group Innovation of Human Movement Care, HU University of Applied Sciences Utrecht, Utrecht, The Netherlands

**Keywords:** Exercise, Behavior, PREVENTIVE MEDICINE, STROKE MEDICINE, Cardiovascular Disease, SLEEP MEDICINE

## Abstract

**Introduction:**

Striking the balance in 24-hour movement behaviour (sedentary behaviour, physical activity and sleep) is expected to reduce the risk of a new major cardiovascular event or death (MACE). We aim to determine the effectiveness and cost-effectiveness of the RISE (*Reduce and Interrupt sedentary behaviour using a blended behavioural intervention to Empower people at risk towards sustainable 24-hour movement behaviour change*) intervention by improving 24-hour movement behaviour for prevention of MACE and gaining quality-adjusted life years (QALYs) in community-dwelling people at risk with a first-ever stroke.

**Methods and analysis:**

This assessor-blinded multicentre randomised controlled trial includes about 1000 participants with a first-ever stroke, of which 752 participants require secondary prevention based on their 24-hour movement behaviour. Participants will be randomly assigned to the experimental group (RISE intervention + usual care) or control (usual care) group. RISE is a 15-week blended care intervention: primary care physiotherapists coach people in their home setting using behaviour change techniques and the RISE eCoaching system. This system consists of: (1) an activity monitor, (2) a smartphone application that provides real-time feedback and contains e-learning modules and (3) a monitoring dashboard for the physiotherapist. A close relative of the participant is involved during the intervention to provide social support. The primary outcome is the effectiveness of the RISE intervention regarding the prevention of MACE measured at one year post randomisation using survival analysis comparing the experimental and control groups. Secondary outcomes include cost-effectiveness for MACE prevention and QALYs and changes in 24-hour movement behaviour over time using compositional data analysis.

**Ethics and dissemination:**

Ethical approval is obtained from Medical Ethics Review Committee Utrecht, NedMec NL83940.000.23. Findings will be disseminated through international peer-reviewed journals and conferences. A sustainable 24-hour movement behaviour change is needed to gain long-term benefits of lowering MACE in patients with stroke. The RISE intervention offers this foundation by integrating behaviour change techniques, the RISE eCoaching system, involvement of participatory support and extensively trained RISE physiotherapists. Consequently, the RISE intervention is expected to be (cost-)effective compared with usual care, and hence, this study will offer a foundation for implementing the RISE intervention in standard poststroke care.

**Trial registration number:**

NCT06124248.

**STRENGTHS AND LIMITATIONS OF THIS STUDY**

- The RISE (*Reduce and Interrupt sedentary behaviour using a blended behavioural intervention to Empower people at risk towards sustainable 24-hour movement behaviour change)* intervention integrates behaviour change techniques, the RISE eCoaching system, participatory support and extensively trained physiotherapists to promote sustainable movement behaviour change.
- All RISE physiotherapists will complete a 4-day course and participate in the RISE learning community (where RISE-related knowledge or cases will be shared during 3-monthly booster sessions) to ensure RISE intervention quality.
- The RISE intervention will be delivered in the home setting and is aligned with the existing healthcare system, enhancing its potential for future implementation within usual care.
- The use of repeated objective measurements will increase statistical power of the study.
- To address the potential limitation that individuals with limited digital health literacy may struggle to engage with the intervention, the application was co-designed with people with stroke to enhance usability, and participants receive coaching on how to use the RISE eCoaching system.

## Introduction

 Globally, stroke is the greatest contributor to the burden of disease, characterised by a high number of disability-adjusted life years.^1^ The combination of a rising stroke incidence and improvements in acute care has led to a higher number of people living with less residual symptoms after stroke.^2^ However, these people are still at risk of a new major cardiovascular event or death (MACE). A quarter of the people will have a MACE within the first year after stroke.^3^ On top of that, the societal costs incurred by a Dutch stroke patient are high, averaging €29 484 in the first year post stroke.^4^ This stresses the importance of secondary prevention in stroke patients who are at risk for MACE from both the patients’ and societal perspective.

Known risk factors for MACE include an elevated systolic blood pressure, a high body mass index and unhealthy lifestyle factors, such as unhealthy 24-hour movement behaviour (sleep, sedentary behaviour (SB) and physical activity).^5 6^ SB is defined as any waking behaviour with an energy expenditure of ≤1.5 metabolic equivalents, while being in a sitting, lying or reclining posture.^7^ There is increasing evidence that suggests that high levels of SB are strongly adversely associated with cardiometabolic health.^8^ Since the amount of time spent in one behaviour will directly modify the time spent in another, research and interventions should integrate this 24-hour movement behaviour.^9^

Due to its multifactorial nature, changing the 24-hour movement behaviour is a challenging task.^10^ The personal situation, including physical or cognitive capabilities and the (social) environment, varies per individual and therefore requires personalised coaching to enable behavioural change.^10^ Behavioural change is highly needed, since our cohort study showed that 79% of the stroke population was highly sedentary (>9.5 hours) and spent minimal time in moderate to vigorous physical activity (MVPA).^11^ A recent review showed that there are no effective interventions to support community-dwelling stroke patients in reducing SB.^12^ To the best of our knowledge, there are no interventions focusing on specifically targeting the 24-hour movement behaviour in people with stroke.

Given the above, our research group developed the RISE (*Reduce and Interrupt SB using a blended behavioural intervention to Empower people at risk towards sustainable 24-hour movement behaviour change*) intervention, primarily focusing on interrupting SB with at least light physical activity (LPA).^13 14^ The RISE intervention is a blended behavioural intervention, using face-to-face coaching by a physiotherapist (behaviour change techniques) combined with activity monitoring and a smartphone application. As part of the RISE intervention, participants receive social support from someone out of their social network who joins them in the intervention to provide participatory support (PS). Our recent pilot study showed promising results regarding the RISE intervention’s feasibility and preliminary effectiveness, as well as the value of adding PS.^14^

Based on input from healthcare professionals and participants from our RISE pilot study, the RISE intervention and preintervention training of healthcare professionals has been optimised.^10^ In this assessor-blinded multicentre randomised controlled trial, the primary objective is to assess the effectiveness of the RISE intervention to prevent MACE after 1 year compared with usual care according to national stroke guidelines.^15^ Secondarily, the cost-effectiveness of the RISE intervention for MACE prevention and quality-adjusted life years (QALYs) and the effect of the RISE intervention on changes in the 24-hour movement behaviour will be analysed.

## Methods

**Figure 1:**
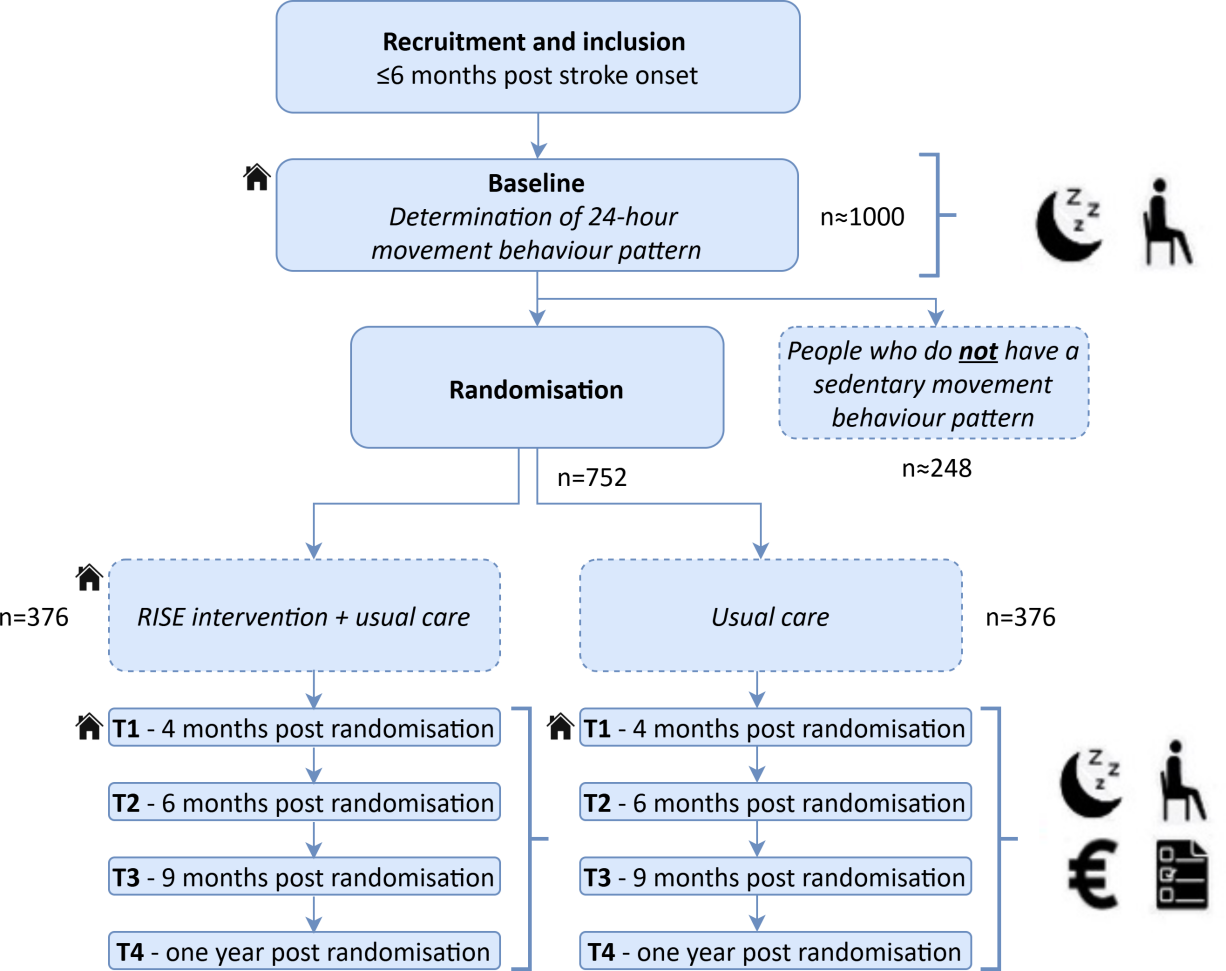
Flowchart of the RISE trial. Until one year post randomisation, occurrence of MACE, cost-effectiveness and 24-hour movement behaviour will be measured. All activities with the icon of a house will take place at the participants’ home.

In this assessor-blinded multicentre randomised controlled trial, participants who require secondary prevention based on their 24-hour movement behaviour (further explained at ‘Study procedures’) will be included and randomly assigned to either the experimental or control group (allocation ratio 1:1) (figure 1). For the protocol, the Standard Protocol Items: Recommendations for Interventional Trials checklist was used, including trial registration details (table 1).

**Table 1:** Trial registration data

Data category	Information
Primary registry and trial identifying number	ClinicalTrials.govNCT06124248
Date of registration in primary registry	08 November 2023 earliest version24 September 2024 last update posted
Secondary identifying numbers	NL83940.000.23 (Medical Ethics Review Committee Utrecht NedMec)
Source(s) of monetary or material support	This work is supported by Dutch Research Council (NWO), Nationaal Regieorgaan Praktijkgericht Onderzoek SIA grant number RAAK-PRO04.093 and Care Research Netherlands & Medical Sciences ZonMW grant number 0930012310010.
Primary sponsor	University Medical Centre Utrecht
Secondary sponsor(s)	N.A.
Contact for public queries	rise@umcutrecht.nl
Contact for scientific queries	rise@umcutrecht.nl
Public title	RISE intervention: heading to sustainable movement behavioural change in people with stroke
Scientific title	Secondary prevention by striking the balance in 24-hour movement behaviour by empowering people at risk with a stroke: rationale and design of the RISE intervention randomised controlled trial
Countries of recruitment	The Netherlands
Health condition(s) or problem(s) studied	Stroke
Intervention(s)	Experimental group: RISE intervention and usual care. The RISE intervention is a 15-week blended care intervention: primary care physiotherapists coach people in their home setting using behaviour change techniques and the RISE eCoaching system. This system consists of: (1) an activity monitor, (2) a smartphone application that provides real-time feedback and contains e-learning modules and (3) a monitoring dashboard for the physiotherapist. A close relative from the participant is involved during the intervention to provide social support.
	Control group: usual care
Key inclusion and exclusion criteria	Ages eligible for study: ≥18 years. Sexes eligible for study: both.Accepts healthy volunteers: no
	Inclusion criteria: if people are aged ≥18 years, had a first-ever symptomatic stroke (excluding transient ischaemic attacks) within 6 months before inclusion, are able to walk independently (functional ambulant category ≥3), were independent regarding activities of daily living pre-stroke (Barthel Index >18), are not participating in a physical rehabilitation programme lasting ≥3 months and are discharged to the home-setting.
	Exclusion criteria: if people are unable to understand the Dutch language or have severe comorbidities that withhold them from safely reducing and interrupting their sedentary time
Study type	Interventional
	Allocation: assessor-blinded multicentre randomised controlled trial
Date of first enrolment	November 2023
Target sample size	1000
Recruitment status	Recruiting
Primary outcome(s)	Effectiveness of the RISE intervention regarding prevention of MACE measured at 1 year post randomisation comparing experimental and control groups.
Key secondary outcomes	Cost-effectiveness of the RISE intervention for MACE prevention and quality adjusted life years.Changes in 24-hour movement behaviour (sedentary behaviour, physical activity and sleep) over time between the experimental and control group.

MACE, new major cardiovascular event or death; N.A., not applicable; RISE, Reduce and Interrupt sedentary behaviour using a blended behavioural intervention to Empower people at risk towards sustainable 24-hour movement behaviour change.

### Participants

Participants will be recruited via stroke units in about 30 hospitals in the Netherlands, the general practitioner (GP) clinic or open recruitment between November 2023 and December 2025. They will be approached post discharge in the hospital or via a GP’s letter and asked for permission to share their contact details with the researchers. Open recruitment will occur via articles and flyers, encouraging interested individuals to contact the researchers directly. With participants’ consent, their eligibility will be assessed by consulting the healthcare professional in charge. The number and reasons for non-participation will be recorded.

Participants will be eligible for participation if they are aged ≥18 years, had a first-ever symptomatic stroke (excluding transient ischaemic attacks (TIA)) within 6 months before inclusion, are able to walk independently (functional ambulant category ≥3), were independent regarding activities of daily living pre-stroke (Barthel Index>18), are not participating in a physical rehabilitation programme lasting ≥3 months and are discharged to the home-setting. Participants will be excluded when they are unable to understand and speak the Dutch language or have severe comorbidities that withhold them from safely reducing and interrupting their sedentary time.

### Study procedures

After obtaining informed consent (online supplemental file 1), baseline measurement (T0) takes place at the participants’ home (figure 1). During this visit, an activity monitor will be provided to objectify the 24-hour movement behaviour. If participants require secondary prevention, it will be based on their 24-hour movement behaviour pattern^11^: *sedentary prolongers,* ≥9.5 hours of sedentary time per day, >50% of the sedentary time is spent in bouts >30 min and not reaching the physical activity guideline (150 min MVPA during the week); *sedentary movers,* <9.5 hours of sedentary time and (1) ≤50% of the sedentary time spent in bouts >30 min or (2) not reaching the physical activity guideline.^16 17^ Randomisation will take place for *sedentary prolongers* and *sedentary movers,* with stratification according to these two profiles. All other participants will be excluded after baseline measurement.

Concealment of allocation will be ensured by individual randomisation by an independent researcher. Randomisation to either the experimental or control group will be done using a computer-generated random sequence with block sizes of two, four and six (Castor Electronic Data Capture 2021, Ciwit B.V., Amsterdam, The Netherlands).

The experimental group receives the RISE intervention from a trained primary care physiotherapist for 15 weeks. Follow-up measurements take place at the end of the intervention period (T1) (ie, 4 months post randomisation) and at 6 months (T2), 9 months (T3) and 12 months post randomisation (T4) (figure 1). Due to the nature of the intervention, participants and primary care physiotherapists will not be blinded for treatment allocation. The researcher who performs the measurements at T1 is blinded to treatment allocation.

### Experimental (RISE intervention + usual care) group

Participants in the experimental group receive usual care, according to national guideline ‘diagnostics, treatment and care for participants with a stroke’ and the RISE intervention.^18^

The RISE intervention is based on the behaviour change wheel (BCW), a method guiding the development of behaviour change interventions.^19^ Extensive explanation on the development of the RISE intervention and the (co-)design process can be found elsewhere.^13^ Preliminary effectiveness and feasibility of the RISE intervention have been determined in a randomised multiple baseline study.^14^ After the pilot study, minor refinements have been implemented in the current study: an updated version of the RISE device is used and the user interface of the RISE app was updated based on participants’ input.

**Figure 2:**
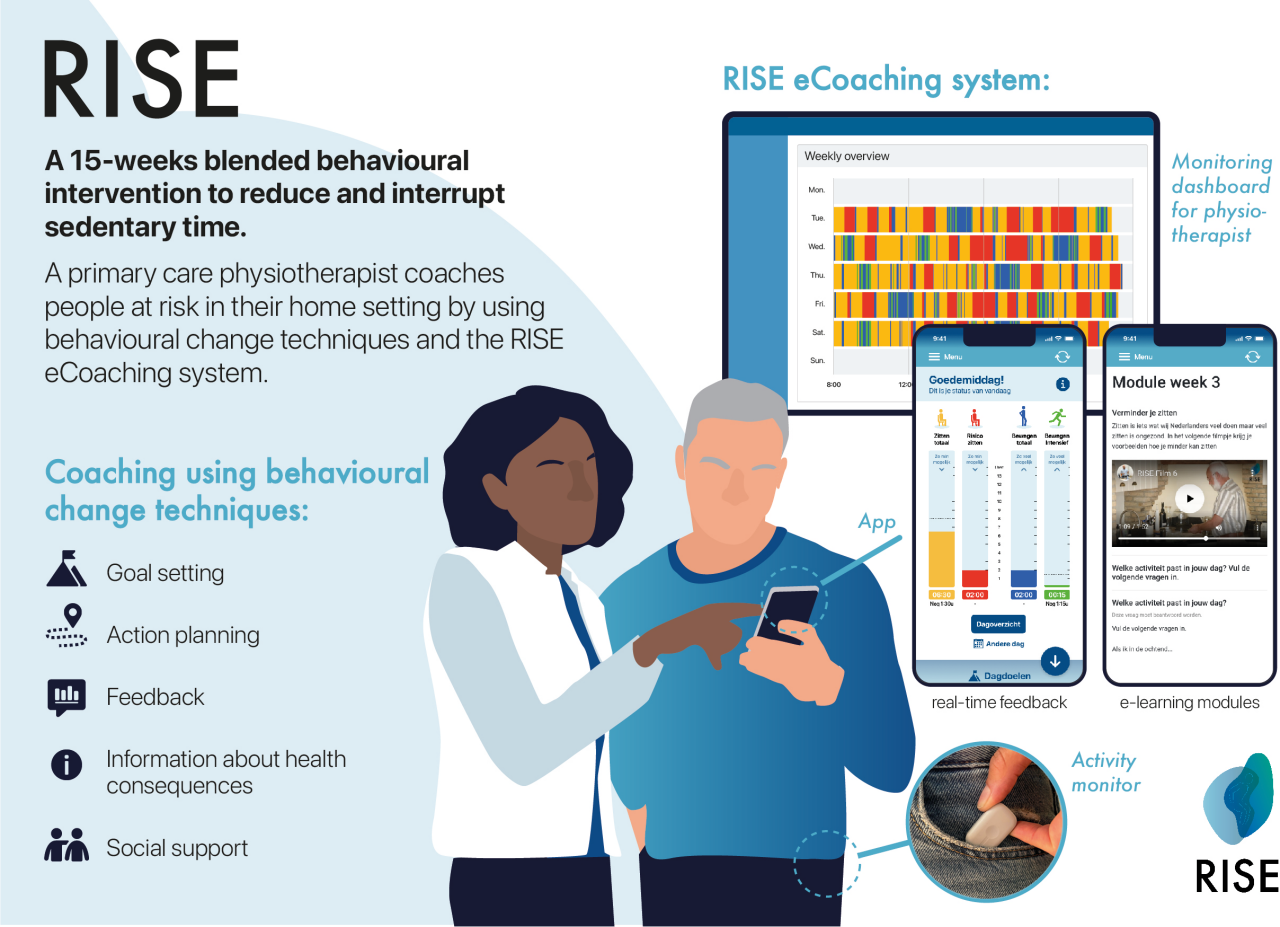
RISE eCoaching system consists of: (1) an activity monitor, (2) a smartphone application that provides real-time feedback and contains e-learning modules and (3) a monitoring dashboard for the physiotherapist.

The RISE intervention is a 15-week blended behavioural intervention, in which a trained primary care physiotherapist coaches participants to reduce and interrupt their sedentary time (figure 2). Physiotherapists provide personalised coaching to people with a stroke in their home setting by using behaviour change techniques and the *RISE eCoaching system* (table 2). The RISE eCoaching system consists of: (1) an activity monitor, (2) a smartphone application that provides real-time feedback and contains e-learning modules and (3) a monitoring dashboard for the physiotherapist. The e-learnings consist of weekly videos about self-management provided with assignments and text. Topics are: lifestyle factors, movement behaviour including SB, examples of reducing SB, participant expectations, action planning, coping planning, PS, a set-back and prevention plan. The intervention is protocolised per week (table 2) and personalised for each participant’s goals, action plans, coping strategies and setbacks. The RISE eCoaching system provides insights into 24-hour movement behaviour and facilitates goal setting and personalised feedback. It recommends goals based on past movement behaviour and graded activity principles, which the physiotherapist and participant finalise together, appearing in the RISE app.^20^ Participants monitor their progress and receive tailored feedback based on their actual movement behaviour compared with their goals (figure 2). Physiotherapists also offer sleep improvement tips if needed.

**Table 2:** Overview protocol RISE intervention

Week	Mode of delivery	Main topic	Elements	Behaviour change techniques^21^
1	At participants’ home (meeting 1)	Introduction of the intervention	Intake Information about the intervention and the purpose and importance of healthy movement behaviour Start self-monitoring	Information about health consequences
	eCoaching	Benefits of reducing sedentary behaviour (1)		Information about health consequences
2	At participants’ home (meeting 2)	Behavioural diagnosis	Coaching sessions to identify facilitators and barriers Discussing the movement behaviour	Goal-settingFeedback on behaviourGraded taskNon-specific reward
	eCoaching	Benefits of reducing sedentary behaviour (2)		Information about health consequences
3	At participants’ home (meeting 3)	Self-monitoring	Discussing movement behaviour Discussing own beliefs about reducing sedentary behaviour Discussing possible barriers Setting cues	Goal-settingFeedback on behaviourHabit reversalReview behavioural goalsBehaviour substitutionPrompts and cuesProblem-solving
	eCoaching	What is self-monitoring?		Feedback on behaviour
4	At participants’ home/online/at physiotherapy practice (meeting 4)	Action planning and goal-settingSleep check	Goal-setting and monitoring Self-monitoring Discussing sleep hygiene rules Developing a strategy to sit less	Goal-settingFeedback on behaviourHabit formationDemonstration of the behaviourInstructions on how to perform the behaviour
	eCoaching	Prompts and cues		Prompts and cues
5	At participants’ home/online/at physiotherapy practice (meeting 5)	Building social support	Discussing the movement behaviour Creating an action plan Discussing the possibilities for social support	Action planningGraded tasksSocial support (unspecified)Social support practicalReview behavioural goals
	eCoaching	Maintenance task self-efficacy		Habit formation
6	At participants’ home/online/at physiotherapy practice (meeting 6)	Increasing self-confidence for reducing sedentary behaviour	Reviewing movement behaviour of past week Providing positive feedback Discussing successes Discussing action plan Discussing social environment Reducing cues	Action planningFeedback on behaviourSocial support (unspecified)Social support practicalGraded task
7	eCoaching	Review own behaviour and the social and physical environment		Review behavioural goals
8	At participants’ home/online/at physiotherapy practice (meeting 7)	Introduction of setbacks	Discussing past week Reinforcing successes Discussing action plan Making an action plan to rearrange physical environment	Feedback on behaviourAction planningRestructuring the physical environmentFocussing on past successes
9	eCoaching	Habit formation and coping planning		Habit formation
10	At participants’ home/online/at physiotherapy practice (meeting 8)	Habit formation and setbacks	Reflection on process so far Feedback on behaviour by participant Reflecting on previous action plan Discussing habits and setbacks Setting a challenging goal and action plan	Feedback on behaviourAction planningFocussing on past successesHabit formation
11	eCoaching	Sustainable change		Behaviour substitution
12	At participants’ home/online/at physiotherapy practice (meeting 9)	Self-monitoring	Discussing past weeks Discussing action plan Discussing pros and cons of reducing sedentary time Discussing activity monitor for after the intervention period Discuss sleep hygiene rules	Feedback on behaviour Action planningReview behavioural goalsGraded task
13–14	eCoaching	Specific action and coping plan for the futureSleep		Discrepancy between current behaviour and goal
15	At participants’ home/online/at physiotherapy practice (meeting 10)	Future proof	Review movement behaviour Addressing remaining questions Action plan for future Closing	Review behavioural goalsAction planningCommitmentBehaviour substitutionValued self-identityFocus on success

Participants will receive *PS* from someone from their social network (eg, a partner or close friend) who joins them in the intervention. The PS will attend all RISE sessions and will use their own RISE eCoaching system. Our pilot study showed that the group with PS had a greater reduction in sedentary time than the group without PS.^14^

The intervention will be delivered by ±130 primary care *RISE physiotherapists* specialised in neurological care. All physiotherapists will be extensively trained in four sessions of 3–4 hours focused on behaviour change techniques, the RISE protocol and motivational interviewing. The training is accredited by the Royal Dutch Society for Physiotherapy. After the training, physiotherapists will join an online learning community, where RISE-related questions or cases will be shared during 3-monthly booster sessions. Physiotherapists make use of the following behaviour change techniques: goal-setting (on behaviour and outcome), action planning, social support, self-monitoring of behaviour, feedback on behaviour, discrepancy between current behaviour and goals, information about health consequences, problem-solving, restructuring the social environment, prompts and cues, habits formation and instructions how to perform the behaviour^21^ (table 2).

### Control group (usual care)

There will be no restrictions for receiving other care as usual. Participants in the experimental and control group receive usual care according to national stroke guidelines.^18^ At T1–T4, participants are asked about all of their healthcare utilisation via the institute for Medical Technology Assessment (iMTA) Medical Consumption Questionnaire (iMCQ) (^22^).

### Outcome measurements

An overview of all the outcome measurements obtained and the accompanying time points is given in table 3. A timeline for the follow-up measurements is provided in figure 1.

**Table 3:** Summary of outcome measurements

	Parameter	Measurement instrument	T0	T1*4 months*	T2*6 months*	T3*9 months*	T4*12 months*
Primary	Major adverse cardiovascular event	Medical records from GP		X	X	X	X
Secondary	Healthcare utilisation	iMTA Medical Consumption questionnaire^22^		X	X	X	X
	(Unpaid) productivity losses	iMTA Productivity Cost questionnaire^22^		X	X	X	X
	Quality of life	Euro Quality of Life 5 Dimensions^26^	X	X	X	X	X
	24-hour: sedentary behaviour	ActivPAL^59^	X	X	X		X
	24-hour: physical activity	ActivPAL	X	X	X		X
	24-hour: sleep	Emfit QS sleep tracker^31^	X	X	X		X
Tertiary	Physical functioning	Short Performance Physical Battery^30^	X	X			
	Sleep quality	Pittsburgh Sleep Quality Index^32^	X	X	X		X
	Level of fatigue	Fatigue Severity Scale-7^33^ and Visual Analogue Scale^33^	X	X	X		X
	End-user satisfaction*	End-User Computing Satisfaction^35^	X	X			
	Feasibility*	Post-Study System Usability Scale^36^		X			
	Acceptability*	System Usability Scale^37^		X			
	Compliance†	Compliance questionnaire		X			
	Self-efficacy	Self-Efficacy for Symptom Management Scale^34^	X	X	X		X
	Self-management skills	Patient Activation Measure^28^	X	X	X		X
Other	Stroke impact	Stroke Impact Scale – Physical^38^	X	X	X		X
	Mental health	Hospital Anxiety and Depression Scale^39^	X	X	X		X
	Comorbidities	Cumulative Illness Rating Scale^40^	X				
	Body Mass Index	Centimetre and scale	X	X			
	Walking speed	5 Metre Walking Test^41^	X	X			
	Blood pressure	Blood pressure monitor	X	X			
	Cognitive functioning	Montreal Cognitive Assessment^60^	X				
	Patient characteristics‡	Intake questionnaire	X				
	Stroke characteristics§	Intake questionnaire	X				
	Vascular risk factors¶	Intake questionnaire	X				
	Stroke severity	National Institutes of Health Stroke Scale^42^	X				

T0=baseline measurement, within 6 months post stroke, T1=four months post randomisation, T2=six months post randomisation, T3=nine months post randomisation, T4=one year post randomisation.

* In participants in the experimental group and physiotherapists.

† In physiotherapists and participants in the experimental group.

‡ Patient characteristics: age, gender, education level, living situation (number of persons in the household).

§ Stroke characteristics: type of stroke, side of stroke, time since stroke.

¶ Vascular risk factors: coronary artery disease, atrial fibrillation, diabetes, hypertension, clinical obesity, smoking and alcohol use, hyperlipidaemia.

GP, general practitioner.

### Primary outcome

The effect of the RISE intervention on the number of MACE will be measured based on the 2000 Consensus Conference of the European College of Cardiology and American College of Cardiology criteria.^23^ MACE is a composite of clinical end points of recurrent stroke or TIA, acute coronary events and cardiovascular death.^3^ Stroke recurrence will be diagnosed by acute neurological symptoms and signs and confirmed by MRI. TIAs are defined according to American Heart Association guidelines.^24^ Acute coronary events (myocardial infarction (MI), cardiac revascularisation and hospitalisation with unstable angina) are diagnosed according to the 2000 Consensus Conference of the European College of Cardiology and American College of Cardiology criteria.^23^ Deaths are regarded to be attributable to a cardiovascular cause (fatal MI, fatal stroke (ie, death within 1 month of MI or stroke), sudden death caused by definite coronary artery disease and congestive heart failure) unless a non-cardiac death could be confirmed. Recurrence of MACE will be collected via medical records of the GP. In the Netherlands, where this study takes place, all hospital-based care—including diagnoses, admissions and clinical events—is systematically reported back to the participants’ GP. Additionally, participants are asked in a questionnaire if any MACE occurred. In case of any discrepancy between the GP’s medical record and questionnaire, the researchers will contact the GP to obtain the correct information from the medical professionals involved. Data from the GP’s medical record will remain the primary source of data collection.

### Secondary outcomes

Cost-effectiveness of the RISE intervention will be assessed for MACE prevention and QALYs. QALYs will be based on the participants’ responses to the Euro Quality of Life 5 Dimensions.^25^ These responses will be converted into utility values using the Dutch tariff, after which QALYs will be estimated using linear interpolation between measurement points.^26^ All costs related to the RISE intervention will be considered: that is, costs of the intervention, other healthcare utilisation, informal care, sports equipment and memberships, as well as losses from unpaid and paid work (ie, absenteeism and presenteeism). Intervention costs will be microcosted, meaning that detailed data will be gathered about the number and kinds of resources that are used while providing the RISE intervention, as well as information about their respective unit costs. All other kinds of resource use will be measured at T1–T4, using modified versions of the iMCQ and the iMTA Productivity Cost Questionnaire.^27^ Resource use will be valued in accordance with the Dutch manual for costing studies in healthcare.^22^

Change in 24-hour movement behaviour will be measured in the experimental and control group. The 24-hour (SB, physical activity and sleep) is measured with the ActivPAL (PAL Technologies Ltd, Glasgow, UK).^28^ This monitor is a combination of a triaxial accelerometer and inclinometer, worn on the anterior side of the unaffected thigh and detects if someone is either sedentary (sitting, lying or reclining), standing or walking. The ActivPAL is sealed in a waterproof sleeve and attached to the skin using hypoallergenic tape. Participants will be instructed to wear the ActivPAL for eight consecutive days. The first 24 hours of the data will be removed in order to counteract a possible Hawthorne effect.^29^ A valid day requires at least 10 hours of waking wearing time. Participants having at least three valid measurement days per time point will be used in the analysis.

**Tertiary outcomes** will be physical functioning (Short Performance Physical Battery^30^) at T0 and T1, sleep quantity and quality (Emfit QS sleep tracker^31^ and Pittsburgh Sleep Quality Index^32^ at T0, T1, T2 and T4), level of fatigue (Fatigue Severity Scale-7^33^ and Visual Analogue Scale at T0, T1, T2 and T4), self-efficacy (Self-Efficacy for Symptom Management Scale^34^ at T0, T1, T2 and T4), self-management skills (Patient Activation Measure^28^ at T0, T1, T2 and T4), end-user satisfaction (End-User Computing Satisfaction^35^ at T1), feasibility (Post-Study System Usability Scale^36^ at T1) and acceptability (System Usability Scale^37^ at T1) of the RISE eCoaching system and compliance to the treatment protocol. Compliance to the treatment protocol will be measured at T1 in the experimental group via a compliance questionnaire: (1) the number of people that completed the intervention (with and without missing sessions); (2) the number of participants that missed one or more of the face-to-face sessions, the reasons for missing and the percentage of missed sessions per participant; (3) the amount of sessions the PS was present; (4) the average amount of times participants used the app during the day.

Other outcomes will be stroke impact (Stroke Impact Scale – Physical^38^ at T0 and T1), mental health (Hospital Anxiety and Depression Scale^39^ at T0, T1, T2 and T4), comorbidities (Cumulative Illness Rating Scale^40^ at T0), Body Mass Index (at T0 and T1), walking speed (5-Metre Walking Test^41^ at T0 and T1), blood pressure, cognitive functioning (Montreal Cognitive Assessment at T0), patient characteristics (age, gender, education level, living situation (number of persons in the household) via intake questionnaire at T0), stroke characteristics (type of stroke, side of stroke, time since stroke via intake questionnaire at T0) and vascular risk factors (coronary artery disease, atrial fibrillation, diabetes, hypertension, clinical obesity, smoking and alcohol use, hyperlipidaemia via intake questionnaire at T0) and stroke severity (National of Health Stroke Scale^42^ at T0). All measurement instruments and accompanied time points are given in table 3.

### Sample size

The required number of subjects was calculated using the Lachin and Foulkes Logrank Test module in the PASS 2008 software (Hintze J. PASS 2008, Kayesville, Utah, USA www.ncss.com). The power calculation was performed based on detecting a difference in MACE between the experimental and control group. In line with the effectiveness of cardiac rehabilitation, we expect that the RISE intervention will decrease the number of MACE by 35%.^43^ In the control group, 25% is expected to have MACE in the first year.^3 44^ To not underpower the study, an event rate of 20% in the control group in the first year is used. Participants will enter the study during an accrual period of 1.5 years. Enrolment is assumed to be completed for 50% when 50% of the accrual time has passed. A follow-up period of 1 year is assumed to have a 20% loss to follow from the experimental group and a 20% loss to follow from the control group. A two-sided log rank test with a power of 0.85 and a 0.05 significance level to detect a difference of 0.07 between 0.80 and 0.87 (the proportions surviving the experimental and control group after 1 year) led to an overall sample size of 752 participants (of which 376 are in the experimental group and 376 in the control group).

*Sedentary prolongers* and *sedentary movers* will be the target population of the study and will be randomised to the intervention or control group. From our RISE cohort study, we know that 67% of the participants with stroke were *sedentary prolongers* or *sedentary movers*.^11^ Therefore, we expect around 1000 participants need to be screened during baseline measurement (figure 1). Based on our pilot study, we expect to include 2–3 participants per hospital per month. With 20 participating hospitals, this will lead to the expected number of 762 participants by December 2025.

Our secondary outcome, effectiveness of the RISE intervention on reducing SB, can be assessed in a smaller population. From the RISE pilot study, we know that the target group had an average of 11 hours and 21 min of sedentary time per day, with an SD of 77 min/day.^14^ This means, with a two-sided significance level of 0.05, a power of 80%, that a sample size of 47 people per group is needed to detect a minimal detectable change of 45 min in SB between our groups.^45^ Accounting for a dropout rate of 20%, 59 participants per group will be used for this analysis.

### Statistical analysis

All analyses will be conducted with R statistical software (Version 3.6.1; R Core Team, 2019) and SPSS for Windows, V.27.0 (IBM Corp, released in 2017, IBM SPSS Statistics, Armonk, New York, USA). After determining the amount and patterns of missing data (missing completely at random, missing at random or missing not at random), any missing values will be imputed using ‘multivariate imputation by chained equations’.

Primarily, MACE will be analysed by survival analysis in which the RISE intervention group will be compared with standard care. Cox proportional hazards model will be used, as this provides insights into the effectiveness as well as the effect size of the RISE intervention.

For the economic evaluation, differences in costs and effects will be estimated, while appropriately accounting for ‘baseline imbalances’, ‘correlated costs and effects’, ‘skewed costs and/or effects’, ‘missing data’ and ‘clustered data’, if applicable.^46^ Then, ‘incremental cost-effectiveness ratios’ will be calculated by dividing the estimated difference in costs by those in effects. Uncertainty regarding the RISE intervention’s cost-effectiveness will be graphically illustrated using ‘cost-effectiveness planes’ and ‘cost-effectiveness acceptability curves’. The latter indicates the probability of the intervention being cost-effective compared with usual care for different values of willingness to pay (ie, amount of money decision-makers are willing to pay per unit of effect gained).^47^ To test the robustness of the results, various sensitivity analyses will be performed (eg, complete-case analysis).^48^

The 24-hour movement behaviour consists of sleep quantity, sedentary time, MPVA and LPA. Participants will wear the ActivPAL accelerometer for eight consecutive days during T0, T1, T2 and T4. Daily estimates will be derived from ActivPAL data using the newest R package GGIR.^49^ Within the compositional data analysis (CoDA), SB, MVPA, LPA and sleep quantity are considered as proportions that sum up to 1 (eg, 24 hours).^50^ The raw accelerometry data will be converted into a compositional dataset using the GGIR package. Isometric log-ratios will be used to map the compositional dataset into real-valued coordinates, allowing standard statistical methods to be used. The compositional dataset will be analysed using multivariate analysis of variance (MANOVA) testing overall differences in 24-hour movement patterns between the control and experimental groups. Compositional differences will be calculated via perturbation and visualised using ternary plots.^51^ The usage of CoDa and MANOVA is strongly favoured over traditional analytical techniques when analysing movement behaviours.^52^

### Patient and public involvement

The RISE intervention was developed in cocreation with relevant end-users and stakeholders: people with stroke and their relatives, physiotherapists, (inter)national behavioural and movement behaviour experts, hardware and software developers and technical experts. The co-creation was an iterative process in which the behaviour change wheel for developing behaviour change interventions was followed step-by-step.^19^ The RISE eCoaching system was developed based on the CeHRes road map for designing eHealth technologies.^53^,^53^ Feedback from pilot study interviews with physiotherapists, participants and their PS was directly incorporated into the RISE trial. Design sketches of the RISE eCoaching system were critically reviewed by people with stroke and their relatives. During the current RISE trial, participants and physiotherapists will be interviewed as part of the evaluation and implementation. A panel of experienced experts will be consulted biannually for advice regarding study procedures.^54^

## Ethics and dissemination

The RISE intervention study has been approved by the Medical Ethics Review Committee Utrecht NedMec NL83940.000.23 (Clinicaltrials.gov registration NCT06124248). Study amendments will be notified to the pertinent ethics committee. Informed consent (online supplemental file 1) will be obtained by researchers. Only the study staff will have access to the final dataset containing pseudonymised data. There are no declarations of interest. As soon as all data have been collected and analysed, pseudonymised data will be shared on reasonable request. Findings will be disseminated through international peer-reviewed journals and conferences. End results will be shared with participants and involved parties. Julius Clinical Utrecht is involved as Data Monitoring Committee. All (serious) adverse events related to the intervention or higher will be registered via line listing and sent to the Medical Ethics Review Committee Utrecht NedMec within three months after the end of the study.

## Discussion

This article represents the design of the RISE intervention randomised controlled trial for people with first-ever stroke who require secondary prevention based on their 24-hour movement behaviour. In this study, we will investigate the effectiveness of the RISE blended behaviour intervention in people with a first-ever stroke at risk to prevent major adverse cardiovascular events (ie, recurrent stroke or TIA, acute coronary events and cardiovascular death, MACE). Next to that, the cost-effectiveness of the intervention for MACE prevention and QALYs will be investigated, and changes in 24-hour movement behaviour will be analysed.

In 2017, the WHO launched the programme, Rehabilitation 2030, with a call for action for global rehabilitation needs.^55^ With an increasing number of patients living with the consequences of stroke, the focus in healthcare shifts towards secondary prevention.^15^ Due to this increasing number of patients and the associated costs, healthcare sectors around the world increasingly focus on personalised cost-effective care for people at risk.^4^ The RISE intervention fits within this scope. People at risk receive the intervention at home from a primary care RISE physiotherapist. By means of a personalised focus on healthier 24-hour movement behaviour, we are aiming to prevent the occurrence of MACE in people living with stroke.

A strength of this study is the personalised and behaviour-oriented approach of the RISE intervention. Behaviour is complex, and being physically active is dependent on more factors than solely physical capacities.^19^ Movement behaviour takes place throughout the day and is mainly driven by personal habits and routines. These are directly related to the individual’s social and physical environment.^10^ Based on promising results of our pilot study results regarding PS, participants involve a participatory supporter who joins them in the RISE intervention.^14^ Furthermore, the RISE intervention takes place in the participants’ home, allowing the physiotherapist to take into account all environmental factors related to the 24-hour movement behaviour. Including these societal and environmental elements will contribute to a sustainable movement behavioural change.^56^

Proper training of therapists is essential to minimise deviations from the intended treatment protocol.^57^ Therefore, RISE physiotherapists will be extensively educated during a 4-day course. This is particularly important as the RISE intervention requires a different approach than regular physiotherapy. Current national guidelines are focused on the improvement of disorders, activities and participation within the personal and environmental context, whereas the RISE intervention requires personalised and behaviour-oriented coaching towards sustainable behaviour change.^15^ Therefore, RISE physiotherapists follow an intensive educational programme on personalised coaching, applying behaviour change techniques and motivational interviewing. All RISE physiotherapists become part of the RISE learning community in which adherence to the RISE protocol will be optimised during 3-monthly meetings. Our learning community set-up is based on an effective trial that also showed the importance of proper training and booster sessions.^58^

A limitation of the RISE intervention might be its suitability for people with limited digital literacy skills. Usage of the RISE eCoaching system requires a certain level of digital skills. To overcome this problem as much as possible, the RISE eCoaching system is developed in collaboration with people with stroke to optimise usability of the system. Next to that, RISE physiotherapist coaches participants in the usage of the system during the 15-week intervention period. Another limitation is that the RISE intervention is currently only available in Dutch, and therefore not yet accessible to non-Dutch speakers. Verbal communication is essential for the personalised coaching involved, and due to the practical difficulty of involving physiotherapists fluent in other languages, the choice was made to currently only offer the RISE intervention in Dutch. As a result, non-Dutch speakers were excluded from participation. In future studies, we will explore how to make the intervention accessible to non-Dutch speakers.

A sustainable 24-hour movement behaviour change is needed to gain long-term benefits of lowering cardiovascular events and mortality in patients with stroke. The RISE intervention offers this foundation by integrating behaviour change techniques, the RISE eCoaching system, involvement of PS and extensively trained RISE physiotherapists. Consequently, the RISE intervention is expected to be (cost-)effective compared with usual care, and hence this study will offer a foundation for implementing the RISE intervention in standard poststroke care.

## Supplementary material

10.1136/bmjopen-2024-094894 online supplemental file 1
