# Supplementary material for: Secondary prevention by striking the balance in 24-hour movement behaviour by empowering people at risk with a stroke: rationale and design of the RISE intervention randomised controlled trial
Source: BMJ Open. 2025 Jun 5;15(6):e094894. doi: 10.1136/bmjopen-2024-094894 (PMC12142160; doi:10.1136/bmjopen-2024-094894)
Supplement: online supplemental file 1 [file bmjopen-15-6-s001.pdf]

Participant information for participation in Medical-Scientific Research

UMC Utrecht

The RISE intervention: Sitting Less After a Stroke

Official title: Secondary prevention by striking the balance in sedentary behaviour, physical activity and sleep: (cost-)effectiveness of a behavioral intervention (RISE) to support people after a stroke towards sustainable behavior change.

Dear Sir/Madam,

With this information letter, we ask if you would like to participate in medical-scientific research. Participation is voluntary. To participate, we need your written consent. You are receiving this letter because you had a stroke (either a brain hemorrhage or a brain infarction) and were admitted to the hospital. Before you decide whether to participate, we will explain what the study involves. This letter describes the research, what it means for you, and the advantages and disadvantages. Please read it carefully and decide if you want to participate. If you do, please fill out the form found in Annex C.

Ask Your Questions

You can decide if you wish to participate based on the information in this letter. Also, we recommend:

- Ask questions to the researcher.

- Talk with your partner, family, or friends.

- Ask questions to the independent expert (see Annex A for contact details).

- Visit www.rijksoverheid.nl/mensenonderzoek for more information.

1. General Information

This study is organized by the Department of Rehabilitation, Physiotherapy Science, and Sport at University Medical Center (UMC) Utrecht, in collaboration with Fontys University of Applied Sciences. About 1000 stroke patients are being asked to participate. The Medical Ethics Review Committee (METC NedMec) has approved this research. General information on research can be found on the central government website at www.rijksoverheid.nl/mensenonderzoek.

2. What is the purpose of the study?

You have been in the hospital for a period of time in the past 6 months because you had a stroke. The purpose of this study is to assess the movement behavior (sitting, moving and sleeping) of people after a stroke. In a subset of the participants, we will also investigate whether the RISE intervention can support people to sit less after a stroke. Within this study we will see if this RISE intervention is (cost) effective.

3. What is the background of the study?

After a stroke, the risk of a new stroke or other cardiovascular diseases is increased. The risk of stroke and other cardiovascular diseases is higher if you sit a lot and don't move much. Within this study we want to map the movement behavior of people after a stroke.

We will also investigate whether a new intervention by a physiotherapist (the so-called RISE intervention) can support people to sit less after a stroke. People receiving this RISE intervention will receive both general information about sitting and physical activity and information based on their own movement and sitting behavior. A physiotherapist coaches these people in 10 sessions at home to sit less and move more.

4. How does the investigation proceed?

For a clear representation of the course of the survey, see Appendix B.

Step 1: Mapping out the movement behaviour.

The movement behaviour behaviour of people after a stroke is mapped by measuring your movement and sleep for a week. During one week, we measure how much you sit and move with an activity monitor, and how the quality of your sleep, and sleep duration, is with a sleep monitor.

This is done as follows:

The researcher will make an appointment with you by telephone to visit you at home. At this home visit, the researcher gives you a movement and sleep monitor, and gives instructions on how to use it. The researcher will help you install these monitors. The researcher will ask you questions and do some short tests. The questions concern, for example, your age and education, but also questions related to the stroke. In addition, the examiner measures, for example, how fast you walk and what your blood pressure is.

- In total, this appointment will last about 45 minutes.

- The activity monitor (see image 1) is affixed to your thigh with a plaster. You are allowed to shower while you wear this. The sleep monitor (see image 2) is placed under your mattress and you will not notice this during sleep.

- After one week, you will return the questionnaires and the exercise and sleep monitor with a reply envelope.

The researcher reviews the data and determines whether you qualify for step 2 of the study based on this. If this shows that you already move in a healthy way, the possible RISE intervention will not be of any added value to you. You already have a healthy movement behaviour pattern. You will therefore not be followed up further in the study.

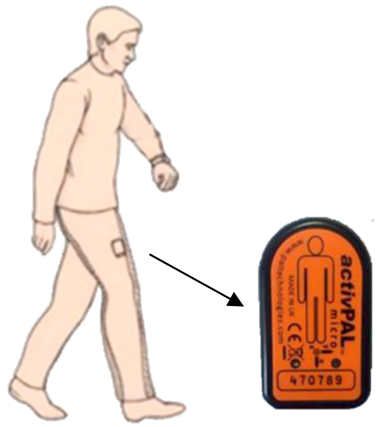

Image 1. Activity monitor attached to the thigh. This image shows the ActivPAL™ monitor (PAL Technologies Ltd, Glasgow, Scotland). Used with permission.

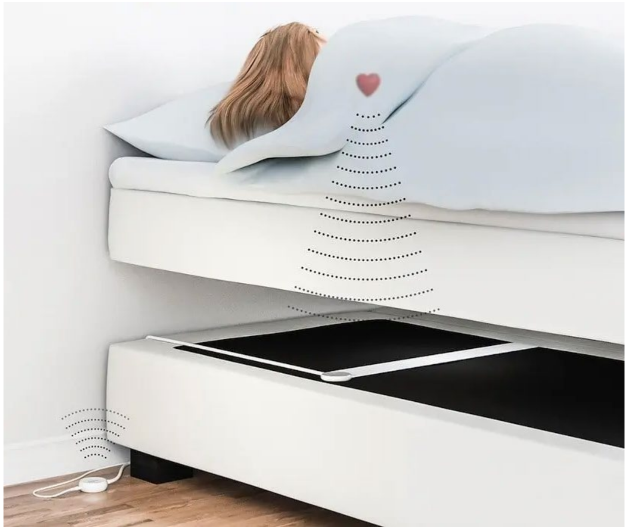

Image 2. Sleep monitor underneath the mattress. This image shows the Emfit QS sleep monitor (Emfit Ltd, Finland). Used with permission.

Step 2: Mapping out the movement behaviour over a longer period of time.

Next, based on their movement behaviour from the baseline measurement, the movement behaviour of a number of individuals will be measured over a longer period of time. This group will be divided by lot into two groups (ratio 1:1, 50% chance of being in one of the two groups). The researchers have no influence on this draw.

1. One group will receive three activity and sleep monitors and four questionnaires over a one-year period. It will take you about 1.5h per measurement to complete the questionnaires. You will wear the activity monitor for one week per measurement. The monitors can be returned by post in a return envelope after each measurement.
2. The other group receives, in addition to the four measurements over a one-year period, an intervention aimed at reducing sitting. Below you can read exactly what this intervention entails:

The aim of the RISE intervention is to sit less and move more.

The RISE intervention is provided by a physiotherapist in your neighborhood. In total, the RISE intervention consists of 10 sessions from the physiotherapist. In the first six weeks, the RISE intervention is once a week, then every other week. In total, RISE intervention lasts 15 weeks. Each appointment lasts 30 minutes. During these visits, where you use an app with your own movement behaviour data, the physiotherapist will coach you to sit less and move more.

The physiotherapist will visit your home for the first three sessions. After that, you will decide together with the physiotherapist whether the remaining RISE interventions will take place at home, online or at the physiotherapy practice. During the physiotherapist's first visit, you will receive an activity monitor to wear in your pocket (see image 3). This device keeps track of how much you sit or move. You can view the results yourself via an app on your smartphone). In addition, the outcomes are discussed with the physiotherapist. Each participant can choose to have a participatory support to join in the RISE intervention, so you can work together towards your goal of sitting less. You can choose who this is and whether you want this.

Appendix B provides an overview of the measurements.

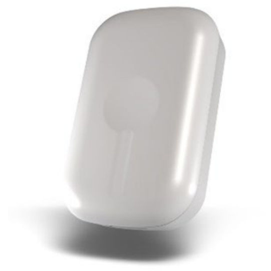

Image 3. The activity monitor worn in the pocket during the RISE intervention. This image shows the RISE device (Activ8 GEN2) by 2M Engineering (Eindhoven, The Netherlands). Used with permission.

5. What is different about participating in this study compared to usual care?

There is not much different in this study compared to usual care. You can receive all the care you would normally receive if you take part. However, we do ask you to write down the care you received so that we can take this into account. We will send you a form to fill in for this purpose.

There is a possibility that when you participate, you will be drawn into the group that receives the RISE intervention, as described in the previous step. If so, you will receive 10 physiotherapy sessions at your home. These physiotherapy treatments are additional to usual care. If you have any questions during treatment, you can ask the physiotherapist. For medical questions, you can ask your general practioner.

6. What arrangements will we make with you?

We want the research to run smoothly. Therefore, we will make the following arrangements with you:

- You will use the activity and sleep monitor in the way explained to you by the researcher.

- In principle, you try to attend every appointment.

- You will contact the researcher in these situations:

- You want to start taking other medicines. Even if these are homeopathic remedies, natural remedies, vitamins or drugstore remedies.
- You are admitted to hospital.
- You suddenly experience problems with your health.
- You no longer wish to participate in the study.
- Your telephone number, address or e-mail address changes.

7. What side effects, adverse effects or discomforts may you experience?

The possible discomforts you may experience by taking part in this study are no different from the possible discomforts you may experience from usual physiotherapy treatment involving increased exercise. These possible discomforts are limited to muscle pain or a short increase in fatigue. However, this is normal and the discomfort usually lasts only one to two days.

8. What are the advantages and disadvantages of taking part in the study?

Taking part in the study can have advantages and disadvantages. We list them below. Think about them carefully and talk about them with others.

It is important to weigh up the possible advantages and disadvantages before you decide to take part. You may benefit from taking part in this study because you will get an insight into your own movement behaviour. Some participants receive only the baseline measurement, some receive the baseline measurement and follow-up measurements, and some receive all measurements and the RISE intervention. The chance of you ending up in the RISE intervention group is about 33%. Treatment may possibly help you to sit less and move more. Less sitting can help reduce the risk of cardiovascular disease. Finally, you will contribute to improving treatment to better support people with stroke in the future.

Participating in the study also means (as aforementioned in section 4, 5 and 6):

- that you spend time taking measurements. Filling in the questionnaires will take about 45 min per measurement.
- wearing the activity monitor may cause slight skin irritation. However, this does not happen very often.
- that you stick to the arrangements.

9. When does the study end?

You decide whether to participate in the study. Participation is voluntary. If you do not want to participate, you will be treated as usual by your healthcare professionals. They will treat you according to the guidelines that apply to the relevant treatments.

If you do participate, you can always change your mind and stop anyway, even during the study. You will then be treated as usual for your stroke. You do not have to say why you are stopping. However, you must tell the researcher immediately. The data collected up to that point will be used for the study.

The researcher will let you know if there is any new information about the study that is important to you. The researcher will then ask you whether you will continue to participate.

In these situations, the study stops for you:

- All visits and measurements as described under point 4 are over
- You choose to stop yourself
- The researcher thinks it is better for you to stop
- University Medical Centre (UMC) Utrecht, the government or the reviewing medical-ethical review committee, decides to stop the study.

You can continue to participate in the study if you are in the treatment group and your participatory support wishes to withdraw from the study.

10. What happens after the study?

The study ends when all participants have finished. After all the data have been processed, the researcher will inform you about the main outcomes of the study, if you wish. This happens about 6 months after the study has ended.

In addition, the researchers may contact your general practitioner to request medical information (e.g. having/not having a second stroke) for up to 5 years after the end of the study.

11. What do we do with your data?

Are you taking part in the study? Then you also give us permission to collect, use and store your data.

For this study, your personal data and some medical data will be used and stored. This includes data such as your name, address, date of birth and data about your stroke and treatment. Some of this data will be exchanged between the physiotherapist involved in the study who gives the treatments and the researchers at the University Medical Centre Utrecht. In addition, your data from the activity monitor and smartphone app user data (including the number of times you use the app) are collected by Appbakkers BV. Based on this, Appbakkers BV can, for example, fix any error messages in the RISE app during the study. Some of the participants will be interviewed by the researcher after the RISE treatment, if applicable. This is done to share experiences about the RISE treatment. If this reveals points of improvement for the app, these will be shared with Appbakkers BV. The collection, use and storage of your data is necessary to answer the questions asked in this survey and publish the results. Data cannot be traced back to individuals. We ask for your consent for the use of your data. In addition, we ask your permission to request medical data from your treating physician and/or general practitioner after the study.

Confidentiality of your data

To protect your privacy, your data will be coded. Your name and other data that can directly identify you are omitted. Only the key to the code allows data to be traced back to you. The key to the code remains safely stored in the University Medical Centre Utrecht. The data cannot be traced back to you in reports and publications about the study either.

Access to your data for verification

Some people at the research site can access all your data. Also to the data without a code. This is necessary to check that the study has been carried out properly and reliably. Persons who can access your data for verification purposes are: the research team and a monitor working for the University Medical Centre Utrecht. They will keep your data confidential. We ask you to give permission for this inspection. The Health Care Inspectorate may inspect your data without your permission. They too will keep your data confidential.

Data retention period

Your data must be kept for 15 years at the research site, the University Medical Centre Utrecht.

Retention and use of data for other research

Your data may also be relevant for other scientific research in the field of sitting and movement after stroke after the end of this study. For this purpose, your data will be kept for 15 years. You can indicate on the consent form whether or not you agree to this. If you do not agree to this, you can simply participate in the current study.

Withdrawal of consent

You can withdraw your consent to the use of your personal data at any time. This applies to this study and also to storage and use for future research. Research data collected up to the moment you withdraw your consent will still be used in the study.

More information on your rights when processing data

For general information on your rights when processing your personal data, please consult the website of the Personal Data Authority (https://autoriteitpersoonsgegevens.nl/nl/onderwerpen/avg-nieuwe-europese-privacywetgeving/controle-over-je-data).

If you have any questions about your rights, please contact the controller of the processing of your personal data. For this study, this is the University Medical Centre Utrecht. See Appendix A for contact details and website.

If you have questions or complaints about the processing of your personal data, we recommend you first contact the researchers or project leader, see Appendix A for contact details. You can also contact the Data Protection Officer of the University Medical Centre Utrecht or the Personal Data Authority.

Registration of the study

Information about this study is also included in a directory of medical-scientific studies namely Clinicaltrials.gov. This does not include data traceable to you. After the study, the

website may show a summary of the results of this study. You can find this study under NL83940.000.23.

12. No compensation for participation

You will not be paid for participating in this study, nor will any travel expenses be reimbursed. For the measurements and treatments, the researcher and physiotherapist will come to you. You will not have to pay for the treatment.

13. Are you insured during the study?

If you participate in the study, you will not run any additional risks. The Medical Ethics Committee NedMec therefore does not require you to take out extra insurance.

14. We will inform your general practitioner if necessary.

The researcher will send your general practitioner an e-mail to let them know that you are participating in the study. This is for your own safety. In case of side findings (e.g. hypertension), the researcher may contact your general practitioner. As a participant, you will also be informed of these side findings.

15. Do you have any questions?

If you have any questions, please contact the research team. For independent advice on joining this study, please contact the independent doctor. She knows a lot about the study but has nothing to do with this study.

If you have any complaints about the study, you can discuss them with the researcher or the physiotherapist from whom you are receiving treatment. If you prefer not to, you can contact the complaints mediators at the University Medical Centre Utrecht. Full details can be found in Appendix A: Contact details.

16. How do you provide consent for the study?

You can first think about this research . Then tell the researcher if you understand the information and whether or not you want to participate. Do you want to participate? Then fill in the consent form that you will find enclosed with this information letter. You and the researcher will both get a signed version of this consent form.

Thank you for your time.

Attachments

A. Contact Details

B. Measurement Overview

C. Consent Form

Attachment A: contact details.

Available upon reasonable request.

Attachment B: Study procedures

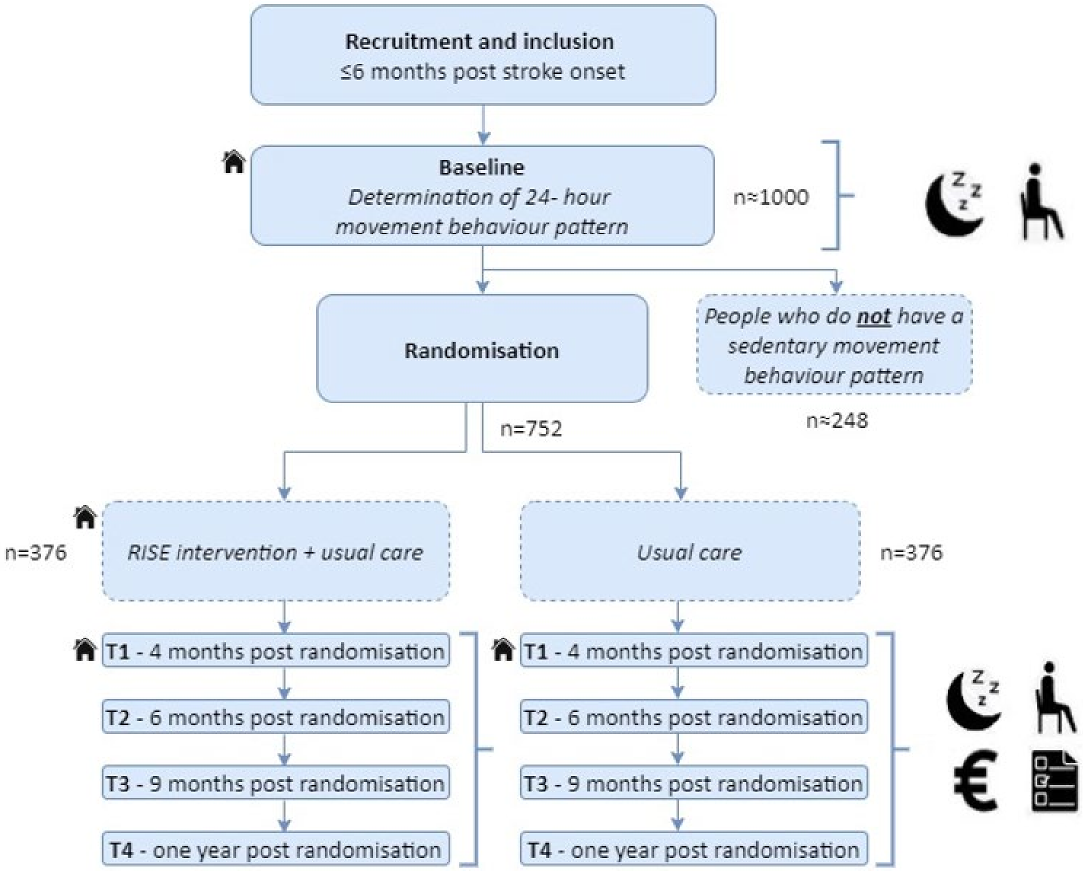

Attachment C:

The RISE intervention for sitting less after stroke

- I have read the information letter. I was also able to ask questions. My questions were answered well enough. I had enough time to decide whether to participate.

- I know that participating is voluntary. I also know that I can decide at any time not to take part in the study after all. Or to quit. I do not then have to say why I want to stop.

- I give the researcher permission to request medical information (e.g. whether or not I will have a second stroke) from my GP after the study.

- I give the researcher permission to contact my GP, in case of side findings.

- I give permission to contact my physiotherapist.

- I consent to the collection and use of my data to answer the research question in this study.

- I give permission to the treating hospital to share my relevant medical data (e.g. degree of stroke severity) for this study. The data must be transferred securely.

- I give my consent to the physiotherapist to share my personal data (with which I can be directly identified) and my treatment data with the researchers at the University Medical Centre Utrecht as part of this study.

- I consent to Appbakkers BV collecting my data from the Activ8 activity monitor and smartphone app.

- I give permission to the University Medical Centre Utrecht to share data on the use of the app with Appbakkers BV. The data will be transferred securely.

- I give permission to keep my data at the research site (University Medical Centre Utrecht) for 15 years after this study.

- I know that for the purpose of monitoring the study, some people may have access to all my data. Those people are listed in this information letter. I consent to such access by these people.

- Would you please tick yes or no in the table below?

I give permission to keep my data for 15 years to use this for other research, as stated in the information letter.	Yes ☐ No☐
I give permission for my personal data to be kept longer and used for future research on sitting and movement after stroke.	Yes ☐ No☐
I consent to being asked if I would like to participate in a follow-up study, if necessary, after this study.	Yes ☐ No☐

- I wish to participate in this study.

My name is (subject): ....

Signature: .... Date : __ / __ / __

I certify that I have fully informed this subject about the said study.

Will any information become known during the study that may affect the subject's consent? If so, I will let this subject know in good time.

Researcher's name (or representative):....................................

Signature:........................... Date:__ / __ / __

Additional information has been given by:

Name:.....................................

Position:...................................

Signature:........................... Date:__ / __ / __

A full information letter will be given to the subject along with a signed version of the consent form.
