# Supplementary material for: Secondary prevention by striking the balance in 24-hour movement behaviour by empowering people at risk with a stroke: rationale and design of the RISE intervention randomised controlled trial
Source: BMJ Open. 2025 Jun 5;15(6):e094894. doi: 10.1136/bmjopen-2024-094894 (PMC12142160; doi:10.1136/bmjopen-2024-094894)
Supplement: Supplementary file 2 [file bmjopen-2024-094894.reviewer_comments.pdf]

PEER REVIEW HISTORY

BMJ Open publishes all reviews undertaken for accepted manuscripts. Reviewers are asked to complete a checklist review form and are provided with free text boxes to elaborate on their assessment. These free text comments are reproduced below.

ARTICLE DETAILS

Title (Provisional)

Secondary prevention by striking the balance in 24-hour movement behaviour by empowering people at risk with a stroke: Rationale and Design of the RISE intervention randomised controlled trial

Authors

Biemans, Camille F.M.; Hartman, Yvonne A.W.; Broers, Suzanne; Pagen, Sophie; Hendrickx, Wendy; Study Group, RISE; van Dongen, Johanna M.; Verschuren, Olaf; English, Coralie; Veenhof, C; Visser-Meilij, Johanna MA; Pisters, Martijn Frits

---

VERSION 1 - REVIEW

**Reviewer**	**1**
**Name**	**Kim, Eunji**
**Affiliation**	**Gachon University Gil Medical Center, Preventive medicine**
**Date**	**21-Jan-2025**
**COI**	**None**

It is a research protocol to investigate the effects of ecoaching intervention in patients with stroke.

I found the research interesting, but have some key comments.

It seems to be an expanded study based on the results of the previous study

(https://www.mdpi.com/2077-0383/13/15/4341). I think that this study should more elaborate the previous study while avoiding the identical figure from the previous study (ex. figure1).

Also, there are many outcomes to investigate, but it seems that it should be described in detail in a more feasible form. For example, in the case of MACE, which is the primary outcome, it is unlikely to be obtained from questionnaires or general practitioners, as they usually visit a high-tier general hospital.

Similarly, how to obtain the cause of death (to distinguish cardiovascular disease) should also be specified not just mentioning it.

The study said that it does not hinder the experimental group from receiving usual precedures, which should be described in more detail than the current sentence ("information on the scope, duration and intensity of treatment will be obtained and taken into account.").

Overall, i reccommed organizing and specifying methods more systematically.

Moreover, beyond mentioning the limitations of this study, it is necessary to describe how to reflect them in the study to overcome.

**Reviewer**	**2**
**Name**	**Mitchell-Miland, Chantele**
**Affiliation**	**VA Pittsburgh Healthcare System University Drive Division**
**Date**	**01-Apr-2025**
**COI**	**None**

Overall, I think this is a very strong study design and has been well thought out. The proposed measures are well-established. I think the abstract needs information including a discussion or conclusion otherwise, the paper looks good.

VERSION 1 - AUTHOR RESPONSE

Reviewer: 1
Dr. Eunji Kim, Gachon University Gil Medical Center
Comments to the Author:
It is a research protocol to investigate the effects of ecoaching intervention in patients with stroke.
I found the research interesting, but have some key comments.

1. It seems to be an expanded study based on the results of the previous study (https://www.mdpi.com/2077-0383/13/15/4341). I think that this study should more elaborate the previous study while avoiding the identical figure from the previous study (ex. Figure 1).

Author's response: Thank you for your valuable comment, and for taking the time to read our previously published pilot study. The current study builds directly on the pilot study, which explored the preliminary feasibility and effectiveness of the RISE intervention. Based on those promising results, we proceeded with a randomized controlled trial using the same intervention, but added a more detailed analysis of 24h movement behaviour, and incorporated a broader range of outcomes and longer follow-up period. As the intervention remained the same, we chose to use the same figure besides some small adjustments. After the pilot study some minor refinements have been implemented. These adjustments (updated version of activity tracker and updated user interface design, based on feedback from participants) are also reflected in the figure in the current study, different than the figure in our pilot study. The figure was specifically developed to briefly present and illustrate the RISE intervention, and we believe it captures its key components. For this reason, we would prefer to retain it in the current study. However, should the editorial board advise otherwise, we are willing to revise or redesign it accordingly. Additionally, we acknowledge the need for more elaboration on the pilot study, and have now included additional information in the methods section.

Author's action:

Page 4 - **Experimental (RISE intervention + usual care) group**

Participants in the experimental group receive usual care, according to national guideline 'Diagnostics, treatment and care for participants with a stroke' and the RISE intervention (18).

The RISE intervention is based on the behaviour change wheel (BCW), a method guiding the development of behaviour change interventions (19). Extensive explanation on the development of the RISE intervention and the (co-)design process can be found elsewhere (13). Preliminary effectiveness and feasibility of the RISE intervention has been determined in a randomized multiple baseline study (14). After the pilot study, minor refinements have been implemented in the current study: an updated version of the RISE device is used, the user interface of the RISE app was updated based on participant's input.

2. Also, there are many outcomes to investigate, but it seems that it should be described in detail in a more feasible form. For example, in the case of MACE, which is the primary outcome, it is unlikely to be obtained from questionnaires or general practitioners, as they usually visit a high-tier general hospital. Similarly, how to obtain the cause of death (to distinguish cardiovascular disease) should also be specified not just mentioning it.

Author's response: Thank you for addressing this topic. In the Dutch healthcare system, we are confident in the feasibility of collecting reliable MACE outcomes due to the structure of medical record-keeping. In the Netherlands, where this study takes place, all hospital-based care—including diagnoses, admissions, and clinical events—is systematically reported back to the patient's general practitioner (GP). In the Netherlands, the GP's medical records are the only location where a complete and longitudinal overview of an individual's medical history is available. For this reason, GP's medical records serve as a reliable source to gather information for outcomes such as MACE. Additionally, participants are asked in a questionnaire if any MACE occurred. In case of any discrepancy between the GP file and patient questionnaire, the researchers will contact the GP to obtain the correct information from the medical professionals involved. Data from the GP will remain the primary source of data collection.

With regard to the classification of cause of death and MACE events, we follow the criteria as defined by the 2000 Consensus Conference of the European Society of Cardiology and the American College of Cardiology (Alpert et al., 2000): *'Deaths are regarded to be attributable to a cardiovascular cause (fatal myocardial infarct, fatal stroke, sudden death caused by definite coronary artery disease, congestive heart failure) unless a non-cardiac death could be confirmed.'* In the Dutch healthcare system, such information is routinely shared with and registered by GP, making the GP's patient records a best available source for identifying and classifying these outcomes. This has now been specified more clearly in the methods section.

Author's action:

Page 7 - **Primary outcome**

The effect of the RISE intervention on the number of MACE will be measured based on the 2000 Consensus Conference of the European College of Cardiology and American College of Cardiology criteria (23). MACE is a composite of clinical end points of recurrent stroke or TIA, acute coronary events, and cardiovascular death (3). Stroke recurrence will be diagnosed by acute neurological symptoms and signs, and confirmed by magnetic resonance imaging. TIAs are defined according to American Heart Association guidelines (24). Acute coronary events (myocardial infarction (MI), cardiac revascularization, hospitalization with unstable angina) are diagnosed according to the 2000 Consensus Conference of the European College of Cardiology and American College of Cardiology criteria (23). Deaths are regarded to be attributable to a cardiovascular cause (fatal MI, fatal stroke (i.e. death within 1 month of MI or stroke), sudden death caused by definite coronary artery disease, congestive heart failure) unless a non-cardiac death could be confirmed. Recurrence of MACE will be collected via medical records of the general practitioner (GP). In the Netherlands, where this study takes place, all hospital-based care—including diagnoses, admissions, and clinical events—is systematically reported back to the patient's GP. Additionally, participants are asked in a questionnaire if any MACE occurred. In case of any discrepancy between the GP's medical record and patient questionnaire, the researchers will contact the GP to obtain the correct information from the medical professionals involved. Data from the GP's medical record will remain the primary source of data collection.

3. The study said that it does not hinder the experimental group from receiving usual procedures, which should

be described in more detail than the current sentence ("information on the scope, duration and intensity of treatment will be obtained and taken into account.").

Author's response: Thank you for your valuable comment. Participants in both the experimental and control group are allowed to receive care as usual, so that the only contrast between groups is the RISE intervention. We understand the need for further clarification on how we obtain information about usual care. Participants in the control group receive usual care according to national stroke guidelines (1). Every three months (T1-T4) participants are asked about all of their healthcare utilization via the iMTA Medical Consumption Questionnaire (iMCQ) (2). The iMCQ is a standardized questionnaire to obtain all healthcare utilization. This allows us to check whether the experimental and control group received comparable usual care, and if necessary, correct for this in the analysis. We have now provided a more detailed description in the methods section.

Author's action:

Page 7 - **Control group (Usual care)**

There will be no restrictions for receiving other care as usual. Participants in the experimental and control group receive usual care according to national stroke guidelines (18). There will be no restrictions for receiving other care as usual. At T1-T4, participants are asked about all of their healthcare utilization via the iMTA Medical Consumption Questionnaire (iMCQ) ((22)).

Overall, I recommend organizing and specifying methods more systematically.

Author's response: In addition, we are grateful for your recommendation to structure the methods section more systematically and in more detail. This prompted us to critically revise the entire methods section. We have added extra references to Table 3 with outcome measures to ensure detailed information. For the same reason, we included further explanations of the content and purpose of both the intake and compliance questionnaires. These changes, made in response to your suggestion, have strengthened the methodological transparency and clarity of the manuscript.

Author's action:

Page 8 - **Tertiary outcomes** will be physical functioning (Short Performance Physical Battery (29)) at T0 and T1, sleep quantity and quality (Emfit QS sleep tracker (30) and Pittsburgh Sleep Quality Index (31) at T0, T1, T2, T4), level of fatigue (Fatigue Severity Scale-7 (32) and Visual Analogue Scale at T0, T1, T2, T4), self-efficacy (Self-Efficacy for Symptom Management Scale (33) at T0, T1, T2, T4), self-management skills (Patient Activation Measure (34) at T0, T1, T2, T4), end-user satisfaction (End-User Computing Satisfaction (35) at T1), feasibility (Post-Study System Usability Scale (36) at T1), and acceptability (System Usability Scale (37) at T1) of the RISE eCoaching system and compliance to the treatment protocol. Compliance to the treatment protocol will be measured at T1 in the experimental group via a compliance questionnaire: 1) the number of people that completed the intervention (with and without missing sessions); 2) The number of participants that missed one or more of the face to face sessions, the reasons for missing, and the percentage of missed sessions per participant; 3) The amount of sessions the participatory support was present; 4) The average amount of times participants used the app during the day.

Other outcomes will be stroke impact (Stroke Impact Scale – Physical (38) at T0 and T1), mental health (Hospital Anxiety and Depression Scale (39) at T0, T1, T2, T4), comorbidities (Cumulative Illness Rating Scale (40) at T0), Body Mass Index (at T0 and T1), walking speed (5 Meter Walking Test (41) at T0 and T1),
blood pressure, cognitive functioning (Montreal Cognitive Assessment at T0), Patient characteristics (age, gender, education level, living situation (number of persons in the household) via intake questionnaire at T0), stroke characteristics (type of stroke, side of stroke, time since stroke via intake questionnaire at T0) and vascular risk factors (coronary artery disease, AF, diabetes, hypertension, clinical obesity, smoking and alcohol use,

hyperlipidemia via intake questionnaire at T0), and Stroke severity (National of Health Stroke Scale (42) at T0). All measurement instruments and accompanied time points are given in Table 3.

Page 9 - **Table 3.** Summary of outcome measurements

	Parameter	Measurement instrument	T0	T1<br>*4M*	T2<br>*6M*	T3<br>*9M*	T4<br>*12M*
Primary	Major Adverse Cardiovascular Event	Medical records from GP		X	X	X	X
Secondary	Healthcare utilization	iMTA Medical Consumption questionnaire (22)		X	X	X	X
	(Unpaid) productivity losses	iMTA Medical Consumption questionnaire (22)		X	X	X	X
	Quality of life	iMTA Productivity cost questionnaire (22)	X	X	X	X	X
	24-hour: Sedentary behaviour	Euro Quality of Life 5 Dimensions (26)	X	X	X		X
	24-hour: Physical activity	ActivPAL (44)					
	24-hour: Sleep	ActivPAL	X	X	X		X
		Emfit QS sleep tracker (30)	X	X	X		X
Tertiary	Physical functioning	Short Performance Physical Battery (29)	X	X			
	Sleep quality	Pittsburgh Sleep Quality Index (31)	X	X	X		X
	Level of fatigue	Fatigue Severity Scale-7 (32) and Visual Analogue Scale	X	X	X		X
	End-user satisfaction*	End-User Computing Satisfaction (35)	X	X			
	Feasibility*	Post-Study System Usability Scale (36)		X			
	Acceptability*	System Usability Scale (37)		X			
	Compliance *****	Compliance questionnaire		X			
	Self-efficacy	Self-Efficacy for Symptom Management Scale (33)	X	X	X		X
	Self-management skills	Patient Activation Measure (34)	X	X	X		X
Other	Stroke impact	Stroke Impact Scale – Physical (38)	X	X	X		X
	Mental Health	Hospital Anxiety and Depression Scale (39)	X	X	X		X
	Comorbidities	Cumulative Illness Rating Scale (40)	X				
	Body Mass Index	Centimeter and scale	X	X			
	Walking speed	5 Meter Walking Test (41)	X	X			
	Blood pressure	Blood pressure monitor	X	X			
	Cognitive functioning	Montreal Cognitive Assessment (42)	X				
	Patient characteristics**	Intake questionnaire	X				
	Stroke characteristics***	Intake questionnaire	X				
	Vascular risk factors****	Intake questionnaire	X				
	Stroke severity	National Institutes of Health Stroke Scale (43)	X				

T0=baseline measurement, within 6 months post stroke, T1=four months post-randomisation, T2=six months post-randomisation, T3=nine months post-randomisation, T4=one year post-randomisation, GP=general practitioner, M=months. *In participants in the experimental group and physiotherapists **Patient characteristics: age, gender, education level, living situation (number of persons in the household ***Stroke characteristics: type of stroke, side of stroke, time since stroke ****Vascular risk factors: coronary artery disease, AF, diabetes, hypertension, clinical obesity, smoking and alcohol use, hyperlipidemia *****In physiotherapists and participants in the experimental group ******In participants in the experimental and control group.

4. Moreover, beyond mentioning the limitations of this study, it is necessary to describe how to reflect them in the study to overcome.

Author's response: Thank you for this comment. In our manuscript, we discuss two main limitations of the RISE intervention: (1) the required level of digital literacy among participants, and (2) the language barrier for participants who do not speak Dutch. While we explained how we addressed the issue of digital literacy, we acknowledge that we did not describe how we plan to overcome the limitation related to language barriers. This happened because overcoming this limitation is not feasible at this stage of the research, primarily due to practical constraints. We understand that this could cause some confusion. Therefore, we have added an explanation to the discussion section to clarify why overcoming the language barrier is currently not feasible.

Verbal communication is a crucial component of the RISE intervention, as the intervention is based on personalized coaching. If participants and physiotherapists do not speak the same language, this can influence the effectiveness of the intervention as they are not able to communicate properly. Due to the practical challenge of finding physiotherapists fluent in languages other than Dutch, we have made the decision to offer the intervention exclusively in Dutch at this stage. As a result, we have chosen to exclude participants who do not speak Dutch to ensure that the effectiveness of the RISE intervention is not affected by language barriers. In future studies, once we have demonstrated the effectiveness of the intervention, we plan to explore how we can make it accessible to non-Dutch speakers.

This explanation has been added to the discussion section.

Author's action:

Page 12 - A limitation of the RISE intervention might be its suitability for people with limited digital literacy skills. Usage of the RISE eCoaching system requires a certain level of digital skills. To overcome this problem as much as possible, the RISE eCoaching system is developed in collaboration with people with stroke to optimize usability of the system. Next to that, RISE physiotherapist coach participants in the usage of the system during the 15-weeks intervention period. Another limitation is that the RISE intervention is currently only available in Dutch, and therefore not yet accessible to non-Dutch speakers. Verbal communication is essential for the personalized coaching involved, and due to the practical difficulty of involving physiotherapists fluent in other languages, the choice was made to currently only offer the RISE intervention in Dutch. As a result, non-Dutch speakers were excluded from participation. In future studies we will explore how to make the intervention accessible to non-Dutch speakers.

<u>**Reviewer: 2**</u>
Chantele Mitchell-Miland, VA Pittsburgh Healthcare System University Drive Division
Comments to the Author:
Overall, I think this is a very strong study design and has been well thought out. The proposed measures are well-established.

1. I think the abstract needs information including a discussion or conclusion otherwise, the paper looks good.

Author's response: Thank you for your kind remarks. We agree that including a discussion or conclusion to the abstract is important. As the journal provides fixed subheadings for the abstract, namely "Introduction," "Methods and analysis," "Ethics and dissemination," we have incorporated the requested point within the 'Ethics and Dissemination' section.

Author's action:

Page 1 **- Introduction** Striking the balance in 24-hour movement behaviour (sedentary behaviour, physical activity and sleep) is expected to reduce the risk of a new major cardiovascular event or death (MACE). We aim to determine the effectiveness and cost-effectiveness of the RISE intervention by improving 24-hour movement behaviour for prevention of MACE and gaining Quality Adjusted Life Years (QALYs) in community dwelling people at risk with a first-ever stroke.

Methods and analysis This assessor-blinded multicentre randomized controlled trial includes about 1000 participants with a first-ever stroke of which 752 participants require secondary prevention based on their 24-hour movement behaviour. Participants will be randomly assigned to the experimental group (RISE intervention + usual care) or control (usual care) group. RISE is a 15 week-blended care intervention: primary care physiotherapists coach people in their home setting using behaviour change techniques and the RISE eCoaching system. This system consists of 1) an activity monitor, 2) a smartphone application that provides real-time feedback and contains e-

learning modules, and 3) a monitoring dashboard for the physiotherapist. A close relative from the participant is involved during the intervention to provide social support. The primary outcome is effectiveness of the RISE intervention regarding prevention of MACE measured at one year post-randomisation using survival analysis comparing experimental and control group. Secondary outcomes include cost-effectiveness for MACE prevention and QALYs and changes in 24-hour movement behaviour over time using Compositional Data Analysis.

Ethics and dissemination Ethical approval METC Utrecht NedMec NL83940.000.23. Findings will be disseminated through international peer-reviewed journals and conferences. A sustainable 24-hour movement behaviour change is needed to gain long-term benefits of lowering MACE in patients with stroke. The RISE intervention offers this foundation by integrating behaviour change techniques, the RISE eCoaching system, involvement of participatory support and extensively trained RISE physiotherapists. Consequently, the RISE intervention is expected to be (cost-)effective compared with usual care and hence this study will offer a foundation for implementing the RISE intervention in standard post-stroke care.

We hope that these revisions addressed all concerns and clarified our study's rigor and feasibility. We remain committed to any further improvements deemed necessary.

References:

1. Kleindorfer Do Fau - Towfighi A, Towfighi A Fau - Chaturvedi S, Chaturvedi S Fau - Cockroft KM, Cockroft Km Fau - Gutierrez J, Gutierrez J Fau - Lombardi-Hill D, Lombardi-Hill D Fau - Kamel H, et al. 2021 Guideline for the Prevention of Stroke in Patients With Stroke and Transient Ischemic Attack: A Guideline From the American Heart Association/American Stroke Association. 2021(1524-4628 (Electronic)).
2. Hakkaart - van Roijen L, Peeters, S., Kanters, T., van Baal, P., Brouwer, W., Drost, R., Evers, S. M. A. A., van Exel, J., Reckers-Droog, V., Tannaoui, N.-E., Thielen, F., & Wijnen, B. Kostenhandleiding voor economische evaluaties in de gezondheidszorg: Methodologie en Referentieprijzen (Herziene versie 2024). Zorginstituut Nederland. 2024.
